# Data mining and safety analysis of BTK inhibitors: A pharmacovigilance investigation based on the FAERS database

**DOI:** 10.3389/fphar.2022.995522

**Published:** 2022-11-11

**Authors:** Qing Wan, Qiang Li, Xin Lai, Tiantian Xu, Jinfang Hu, Hongwei Peng

**Affiliations:** ^1^ Department of Pharmacy, The First Affiliated Hospital of Nanchang University, Nanchang, China; ^2^ Department of Chemotherapy, Jiangxi Cancer Hospital, Nanchang, China

**Keywords:** BTK inhibitors, safety, data mining, non-proportional analysis, B cell malignancies

## Abstract

**Objective:** The introduction of Bruton’s tyrosine kinase (BTK) inhibitors was a milestone in the treatment of B-cell malignancies in recent years owing to its desired efficacy against chronic lymphocytic leukaemia and small cell lymphocytic lymphoma. However, safety issues have hindered its application in clinical practice. The current study aimed to explore the safety warning signals of BTK inhibitors in a real-world setting using the FDA Adverse Event Reporting System (FAERS) to provide reference for clinical rational drug use.

**Methods:** Owing to the short marketing time of other drugs (zanbrutinib and orelabrutinib), we only analysed ibrutinib and acalabrutinib in this study. All data were obtained from the FAERS database from January 2004 to December 2021. Disproportionality analysis and Bayesian analysis were utilised to detect and assess the adverse event (AE) signals of BTK inhibitors.

**Results:** In total, 43,429 reports of ibrutinib were extracted and 1527 AEs were identified, whereas 1742 reports of acalabrutinib were extracted and 220 AEs were identified by disproportionality analysis and Bayesian analysis. Among reports, males were more prone to develop AEs (58.2% for males vs. 35.6% for females treated with ibrutinib, and 55.9% vs. 31.9%, respectively, for acalabrutinib), and more than 30% of patients that suffered from AEs were over 65 years of age. Subsequently, we investigated the top 20 preferred terms (PTs) associated with the signal strength of ibrutinib and acalabrutinib, and our results identified 25 (13 vs. 12, respectively) novel risk signals. Among the top 20 PTs related to death reports, the terms infectious, pneumonia, pleural effusion, fall, asthenia, diarrhoea, and fatigue were all ranked high for these two BTK inhibitors. Further, cardiac disorders were also an important cause of death with ibrutinib.

**Conclusion:** Patients treated with ibrutinib were more prone to develop AEs than those treated with acalabrutinib. Importantly, infection-related adverse reactions, such as pneumonia and pleural effusion, were the most common risk signals related to high mortality associated with both BTK inhibitors, especially in elderly patients. Moreover, cardiovascular-related adverse reactions, such as atrial fibrillation and cardiac failure, were fatal AEs associated with ibrutinib. Our results provide a rationale for physicians to choose suitable BTK inhibitors for different patients and provide appropriate monitoring to achieve safer therapy and longer survival.

## Introduction

B-cell malignancies (BCMs) include non-Hodgkin’s lymphoma, comprising 93% of all cases, and all types of chronic lymphocytic leukaemia, such as mantle cell lymphoma (MCL), follicular lymphoma, marginal zone lymphoma (MZL), diffuse large B-cell lymphoma, Waldenstrom macroglobulinemia (WM), chronic lymphocytic leukaemia (CLL), small lymphocytic lymphoma, which are the most common haematological malignancies (Swerdlow et al., 2016; Teras et al., 2016). Cancer statistics released by the United States in 2021 estimated that the number of new cases of BCM would reached 102,810 and that the number of deaths would reach 25,040 in 2021 (Siegel et al., 2021). Bruton’s tyrosine kinase (BTK) is a non-receptor kinase that plays a crucial role in oncogenic signalling and is an essential protein for B cell receptor (BCR) signalling that is critical for the proliferation and survival of leukemic cells in many B cell malignancies (Burger and Wiestner 2018; Pal et al., 2018). As a novel agent approved for BCM treatment, BTK inhibitors were proven to have high efficacy for the treatment of haematological malignancies, such as CLL, WM, MCL, and MZL, as well as chronic graft-versus-host disease (Zelenetz et al., 2019; Wierda et al., 2020). The advent of BTK inhibitors was a milestone in BCM treatment, as the chemotherapy-free era was imminent. To date, there are four BTK inhibitors approved in the United States and China, including the first-generation ibrutinib, the second-generation acalabrutinib and zanubrutinib (Table 1), and orelabrutinib, recently approved by the China Food and Drug Administration (CFDA) and released in clinical practice in December 2021. Although the effectiveness of those BTK inhibitors has been confirmed in numerous clinical trials (Novero et al., 2014; Mercier, Janssens, and Maertens 2019; Byrd et al., 2020; Rogers et al., 2021), approximately 10%–20% of patients remain intolerant to BTK inhibitors owing to serious adverse events (AEs) (Wu, Zhang, and Liu 2016; O'Brien et al., 2018; Byrd et al., 2019; Hillmen et al., 2019; Xu et al., 2020). Therefore, it is of great importance to identify and analyse such potential AEs in real-world practice.

**TABLE 1:** Summary of FDA-approved BTK inhibitors.

Generic name	Brand name	Target	Approval year
Ibrutinib	Imbruvica	BTK	2013
Acalabrutinib	Calquence	BTK	2017
Zanubrutinib	Brukinsa	BTK	2019

FDA, US Food and Drug Administration; BTK, Bruton’s tyrosine kinase inhibitor.

It was reported that the incidence of haematotoxicity (above grade 3) induced by ibrutinib is 41% (Woyach et al., 2018). Owing to its high intolerance rate, various novel BTK inhibitors with fewer safety concerns have been developed. However, due to the strict study entry criteria and relatively small sample sizes, the safety figures released in clinical trials have limitations. A real-world study could thus provide more comprehensive information on drug safety. Consequently, we aimed to evaluate current BTK inhibitors based on a large real-world patient population by analysing AEs in the FDA’s Adverse Event Reporting System (FAERS). We also investigated the times to onset and fatality rates associated with different BTK inhibitors, to provide a reference for clinical rational drug use.

## Materials and methods

### Data source and collection

We performed a retrospective pharmacovigilance study using data from the FAERS database covering the period from January 2004 to December 2021. FAERS contains real-world results from a large population and under conditions that might have been overlooked in controlled studies, which lack the ability to detect the whole spectrum of adverse drug reactions. A deduplication procedure was performed according to the FDA’s recommendations, selecting the latest FDA_DT when the CASEIDs were the same and selecting the higher PRIMARYID when the CASEID and FDA_DT were the same. AEs were coded using the Medical Dictionary for Regulatory Activities (MedDRA) (version 25.0) system organ class (SOC) and preferred term (PT) level. The drugs in the FAERS database can be reported using arbitrary drug names; therefore, the MICROMEDEX^®^ (Index Nominum) was utilised as a dictionary for BTK inhibitors (Table 1).

### Data mining

Based on disproportionality analysis and Bayesian analysis, the reporting odds ratio (ROR), the proportional reporting ratio (PRR), the Bayesian confidence propagation neural network (BCPNN), and the multi-item gamma poisson shrinker (MGPS) were used to calculate the association between drugs and AEs. The equations and criteria for the four algorithms are shown in Supplementary Table S1. In this study, AEs were extracted based on the circumstances that one of the aforementioned four indices met the criteria (Evans, Waller, and Davis 2001). Reports with input error (EVENT_DT earlier than START_DT) or an inaccurate date of entry were excluded. The proportion of the SOC was calculated as the number of events at the SOC level divided by the total number of events associated with each BTK inhibitor. In addition, reports with fatal events attributed to drug toxicity were counted, and the fatality rate was calculated as the number of fatal events divided by the total number of related events associated with each BTK inhibitor.

### Data analysis and statistics

Descriptive analysis was used to summarise the clinical characteristics of the patients treated with BTK inhibitors collected from the FAERS database. Data mining and all statistical analyses were performed using MYSQL software (version 8.0).

## Results

### Descriptive analysis

During the study period, 1,44,64,087 total reports were retrieved from the FARES database after deduplication. Among them, 43,429 reports were suspected to be related to ibrutinib and 1,742 reports were suspected to be related to acalabrutinib. For zanubrutinib, owing to its short marketing time, there were only 176 reports suspected to be related to the this drug, making it impossible to analyse the data using such a small sample size. The clinical characteristics of events associated with BTK inhibitors are presented in Table 2. According to the results, approximately 70% indications of BTK inhibitors analyzed in this study were lymphocytic malignancies (Chronic lymphocytic leukaemia, mantle cell lymphoma and waldenstrom’s macroglobulinaemia ranked the top three indications of the two BTK inhibitors induced AEs), which was also in accordance with the clinical practice.

**TABLE 2:** Clinical characteristics of patients at risk of AEs using BTK inhibitors based on the FAERS database (January 2004 to December 2021).

Characteristics	Number of reports, no. (%)	
	Ibrutinib	Acalabrutinib
Country		
United States	33,456 (77.0)	1229 (70.6)
Canada	1267 (2.9)	107 (6.1)
France	1128 (2.6)	19 (1.1)
United Kingdom	788 (1.8)	37 (2.1)
Germany	687 (1.6)	11 (0.6)
Others	6103 (14.1)	339 (19.5)
Reporter		
Medical staff	15,608 (35.9)	707 (40.6)
Non-medical staff	27,754 (63.9)	804 (46.1)
Unknown or missing	67 (0.2)	231 (13.3)
Reporting time		
2013	23 (0.1)	—
2014	1058 (2.4)	—
2015	3413 (7.9)	—
2016	3816 (8.8)	—
2017	4856 (11.2)	18 (1.0)
2018	6385 (14.7)	170 (9.8)
2019	7791 (17.9)	161 (9.2)
2020	9001 (20.7)	451 (25.9)
2021	7086 (16.3)	942 (54.1)
Sex		
Female	15,438 (35.6)	556 (31.9)
Male	25,286 (58.2)	974 (55.9)
Unknown or missing	2705 (6.2)	212 (12.2)
Age (year)		
<18	62 (0.1)	3 (0.2)
18–44	327 (0.8)	6 (0.3)
45–64	4762 (11.0)	180 (10.3)
≥65	17,545 (40.4)	692 (39.7)
Unknown or missing	20,733 (47.7)	861 (49.5)
Indication		
Chronic lymphocytic leukaemia	21,526 (49.57)	778 (44.66)
Mantle cell lymphoma	3573 (8.23)	290 (16.65)
Waldenstrom’s macroglobulinaemia	2771 (6.38)	28 (1.61)
Non-Hodgkin’s lymphoma	859 (1.98)	14 (0.8)
Lymphocytic leukaemia	812 (1.87)	26 (1.49)
B-cell small lymphocytic lymphoma	805 (1.85)	15 (0.86)
Diffuse large B-cell lymphoma	464 (1.07)	11 (0.63)
Others	3837 (8.83)	161 (9.25)
Unknown or missing	8782 (20.22)	419 (24.05)

Abbreviations: FAERS, Food and Drug Administration’s Adverse Event Reporting System; AEs, adverse effects.

The results indicated males were more prone to be affected by AEs than females in both ibrutinib and acalabrutinib (58.2% vs. 35.6%, 55.9% vs. 31.9%, respectively). The age of the majority of patients reporting AEs with ibrutinib or acalabrutinib was ≥65 years (40.4% and 39.7%, respectively). Most AEs were reported from the United States (77.0% and 70.6%, respectively), and the number of AE reports increased year over year. Cases were mainly submitted by non-healthcare professionals (63.9% and 46.1%, respectively) for both ibrutinib and acalabrutinib.

### Disproportionality analysis

**FIGURE 1:**
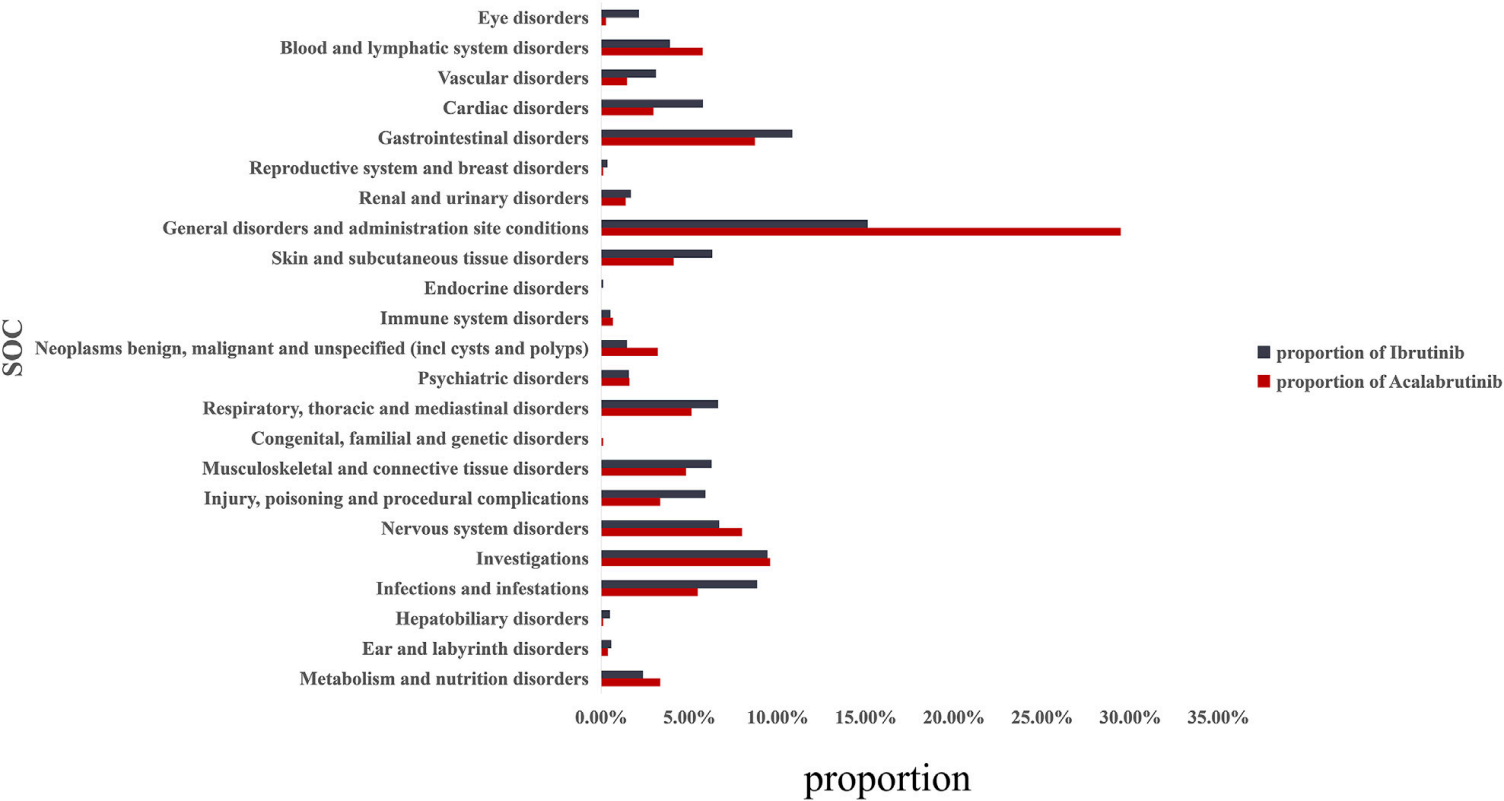
Proportion of BTK inhibitor-related AEs at the System Organ Class (SOC) level. AEs, adverse effects; BTK, Bruton’s tyrosine kinase.

Overall, based on the criteria of the four algorithms, we found 1527 AEs related to ibrutinib and 220 AEs related to acalabrutinib excluding product problems, various injuries, and other irrelevant signals. Table 3 shows the top 20 PTs ordered by signal strength referring to ROR, PRR, BCPNN, and MGPS. Among them, we identified some PTs that were not recorded in the instruction manual of BTK inhibitors as follows: 13 AEs for ibrutinib, including pseudohyperkalaemia (PT: 10052185), prostatic mass (10064022), nail growth abnormal (10064764), blood blister (10005372), immunoglobulins abnormal (10021497), ear neoplasm (10055016), capillary fragility (10007191), prostatic specific antigen decreased (10036972), nail cuticle fissure (10079216), hair texture abnormal (10019049) mastoid effusion (10069008), and scrotal haematocoele (10061517); 12 AEs for acalabrutinib, including oral blood blister (10076590), skin mass (10067868), lymph node pain (10025182), tumour lysis syndrome (10045170), decreased immune responsiveness (10011968), onychoclasis (10048886), melanocytic naevus (10027145), prostatomegaly (10051482), immunodeficiency (10061598), lung opacity (10081792), malignant neoplasm progression (10051398), and Richter’s syndrome (10058728). Figure 1 shows the proportions of SOCs and Supplementary Table S2 shows the signal strengths of SOCs; significant signal overlap emerged for the SOCs of the BTK inhibitors. The proportion of general disorders and administration site conditions (15.1%, 29.5%, respectively), investigations (9.4%, 9.6%, respectively), and gastrointestinal disorders (10.9%, 8.7%, respectively) were all higher than other SOCs for ibrutinib and acalabrutinib. When compared with each other, the proportions of cardiac disorders (5.8%) and eye disorders (2.1%) were relative higher with ibrutinib than with acalabrutinib, whereas blood and lymphatic system disorders (5.8%) and neoplasms benign, malignant, and unspecified (3.2%) were relative higher with acalabrutinib than with ibrutinib.

**TABLE 3:** Top 20 preferred terms (PT) for signal strength.

Ibrutinib						Acalabrutinib					
PT	*N*	ROR (95%two-sided CI)	PRR (χ2)	IC (95%two-sided CI)	EBGM (95% one-sided CI)	PT	*N*	ROR (95%two-sided CI)	PRR (χ2)	IC (95%two-sided CI)	EBGM (95% one-sided CI)
Pseudohyperkalae-mia	5	1660.4 (194, 14,213.3)	1660.3 (1381.9)	44.1 (5.2, 377.5)	523467361935.8 (61153163443.6)	Richter’s syndrome	4	7881.5 (2114.3, 29,380.3)	7860.1 (17,462.5)	39.5 (10.6, 147.2)	9462727139.6 (2538460424.9)
Prostatic mass	6	664.2 (166.1, 2655.9)	664.1 (1324.2)	44.9 (11.2, 179.7)	418773889548.7 (104728347121.5)	Oral blood blister	4	772.7 (278.9, 2140.7)	770.6 (2850.8)	42.1 (15.2, 116.6)	1548446259.2 (558925560)
Mastoid effusion	3	498.1 (83.2, 2981.2)	498.1 (595.3)	43.1 (7.2, 257.9)	376896500593.8 (62973802024.1)	Skin mass	4	205.2 (76.2, 553.2)	204.7 (794.2)	43.9 (16.3, 118.4)	434512980.9 (161216696.2)
Nail growth abnormal	36	291.8 (186.5, 456.6)	291.6 (5550.6)	50.6 (32.4, 79.2)	293685584878.3 (187680362933.1)	Lymph node pain	4	177.5 (66, 477.8)	177.0 (687.8)	44.1 (16.4, 118.8)	376834266.6 (140007155.9)
Blood blister	146	209.7 (170.4, 258)	209 (18,546.6)	54.9 (44.7, 67.6)	242622967754.4 (197204407472.7)	Tumour lysis syndrome	19	163.7 (103.7, 258.4)	161.6 (2984.2)	48.8 (30.9, 76.9)	344575455.9 (218343141.2)
Immunoglobulins abnormal	8	204.4 (84.7, 493.1)	204.3 (1002.1)	46.6 (19.3, 112.4)	239299365456.4 (99178600804.4)	Decreased immune responsiveness	3	154.6 (49.4, 484.3)	154.3 (449.9)	43.5 (13.9, 136.2)	329244372.1 (105129752.4)
Ear haemorrhage^a^	100	195.8 (152.9, 250.7)	195.3 (12,172.8)	53.9 (42.1, 69)	232652160860.4 (181699662242.4)	Onychoclasis	4	137.8 (51.3, 370.1)	137.4 (534.2)	44.5 (16.6, 119.5)	293670842.3 (109322808.4)
Nail bed bleeding^a^	16	171.4 (93.8, 313.5)	171.4 (1787.6)	48.7 (26.7, 89.1)	213841986152.5 (116958710680.9)	Melanocytic -naevus	4	89.8 (33.5, 240.6)	89.5 (347)	45.1 (16.8, 120.9)	192245020.9 (71735647)
Ear neoplasm	8	166.1 (71.1, 388)	166 (874.8)	46.8 (20, 109.3)	209386944774.3 (89606402780.7)	Malignant -neoplasm progression	69	83.7 (65.6, 106.6)	79.8 (5328.6)	53.5 (42, 68.2)	171522287 (134570765.6)
Scrotal haematocoele	6	166 (62.3, 442.4)	166 (656.1)	45.9 (17.2, 122.4)	209386944774.3 (78581686678.9)	Pseudomonal bacteraemia^a^	3	79.4 (25.5, 247.6)	79.2 (229.9)	44.4 (14.3, 138.6)	170329088.5 (54621178.7)
Full blood count abnormal^a^	389	162.5 (143.9, 183.5)	161.1 (41,669.5)	58.0 (51.4, 65.5)	205167560496.8 (181699883256)	Prostatomegaly	3	77.7 (24.9, 242.3)	77.6 (225)	44.5 (14.3, 138.7)	166771300.8 (53485378.5)
Capillary fragility	12	159.4 (80.1, 317.3)	159.4 (1276.1)	48.0 (24.1, 95.5)	203727838158.8 (102349265405.1)	Immunodeficien-cy	4	70.2 (26.2, 188.1)	70.1 (270.4)	45.5 (17, 121.7)	150733706.6 (56300169.2)
Cerebral aspergillosis^a^	24	150.4 (92.9, 243.7)	150.4 (2451)	50.0 (30.9, 81.1)	195790389918.9 (120870358877.4)	Contusion^a^	52	59.6 (45.2, 78.7)	57.6 (2875)	53.1 (40.3, 70.1)	124007512.9 (93955922.1)
Prostatic specific antigen decreased	5	138.4 (48.7, 392.8)	138.4 (481.3)	45.6 (16.1, 129.4)	184753186565.6 (65084642410.7)	Sinus headache^a^	3	56.0 (18, 174.5)	55.9 (160.9)	44.9 (14.4, 140)	120515864.5 (38698730.9)
Skin mass^a^	57	136.3 (100.2, 185.6)	136.2 (5423.8)	52.6 (38.7, 71.7)	182679426308.2 (134197567691.5)	Haemorrhage subcutaneous^a^	3	52.9 (17, 164.8)	52.8 (151.7)	45.0 (14.5, 140.2)	113856342.6 (36566816.4)
Blood iron abnormal	10	132.9 (63.8, 276.6)	132.8 (934.5)	47.6 (22.9, 99.2)	179474524092.3 (86194633637.9)	Full blood count abnormal^a^	6	49.9 (22.4, 111.6)	49.7 (285.2)	47.1 (21.1, 105.3)	107260131.3 (48011250.2)
Nail cuticle fissure	4	120.8 (38.4, 379.3)	120.7 (348.3)	45.1 (14.4, 141.6)	167509555819.5 (53335821793.2)	Lung opacity	4	49.0 (18.3, 130.9)	48.8 (186.4)	46.0 (17.2, 122.9)	105271377.3 (39361127.5)
Renal cyst haemorrhage^a^	5	118.6 (42.7, 329.3)	118.6 (429.6)	45.8 (16.5, 127.1)	165305482716.6 (59538020198.8)	Head discomfort^a^	3	43.8 (14.1, 136.2)	43.7 (124.5)	45.3 (14.6, 141)	94208566.7 (30272516)
Hair texture abnormal	84	108.7 (85, 139.1)	108.5 (6745)	54.0 (42.2, 69.1)	154737566226.2 (120930338214.7)	Skin cancer^a^	7	38.3 (18.2, 80.6)	38.1 (252.1)	47.9 (22.8, 100.9)	82295942.8 (39107374.3)
Dyschezia^a^	10	107.1 (52.5, 218.5)	107.1 (794.8)	47.9 (23.5, 97.6)	153209959591 (75109564045.8)	Blood urine present^a^	8	32.5 (16.2,65.3)	32.4 (242.5)	48.5 (24.2,97.4)	69921629.1 (34861457.3)

^a^ AEs mentioned in the instructions.

Abbreviations: N, the number of reports of BTK-associated AEs; ROR, reporting odds ratio; CI: confidence interval; PRR, proportional reporting ratio; χ2, chi-squared; BTK, Bruton’s tyrosine kinase inhibitor; FAERS, the FDA’s Adverse Event Reporting System.

### Analysis of adverse events associated with Bruton’s tyrosine kinase inhibitors in terms of infection, cardiovascular toxicities, and haemorrhage

Infection, cardiovascular toxicities, and haemorrhage were the most common AEs mentioned in the instruction manuals for BTK inhibitors. Therefore, we performed an analysis of those AEs, and the result is shown in Supplementary Table S3. From the data, even with the same SOC, there were differences in PTs between ibrutinib and acalabrutinib. According to the results, for the SOC of infections and infestations, ibrutinib was mainly centred on fungal infections, such as *Aspergillus* and *Cryptococcosis*; however, acalabrutinib was mainly centred on bacterial infections, such as *Pseudomonas* and *Clostridium difficile.* For the SOC of cardiac disorders, blood, and lymphatic system disorders, ibrutinib seemed to cause more AEs than acalabrutinib based on the data.

### Fatalities due to Bruton’s tyrosine kinase inhibitor-associated adverse events

**FIGURE 2:**
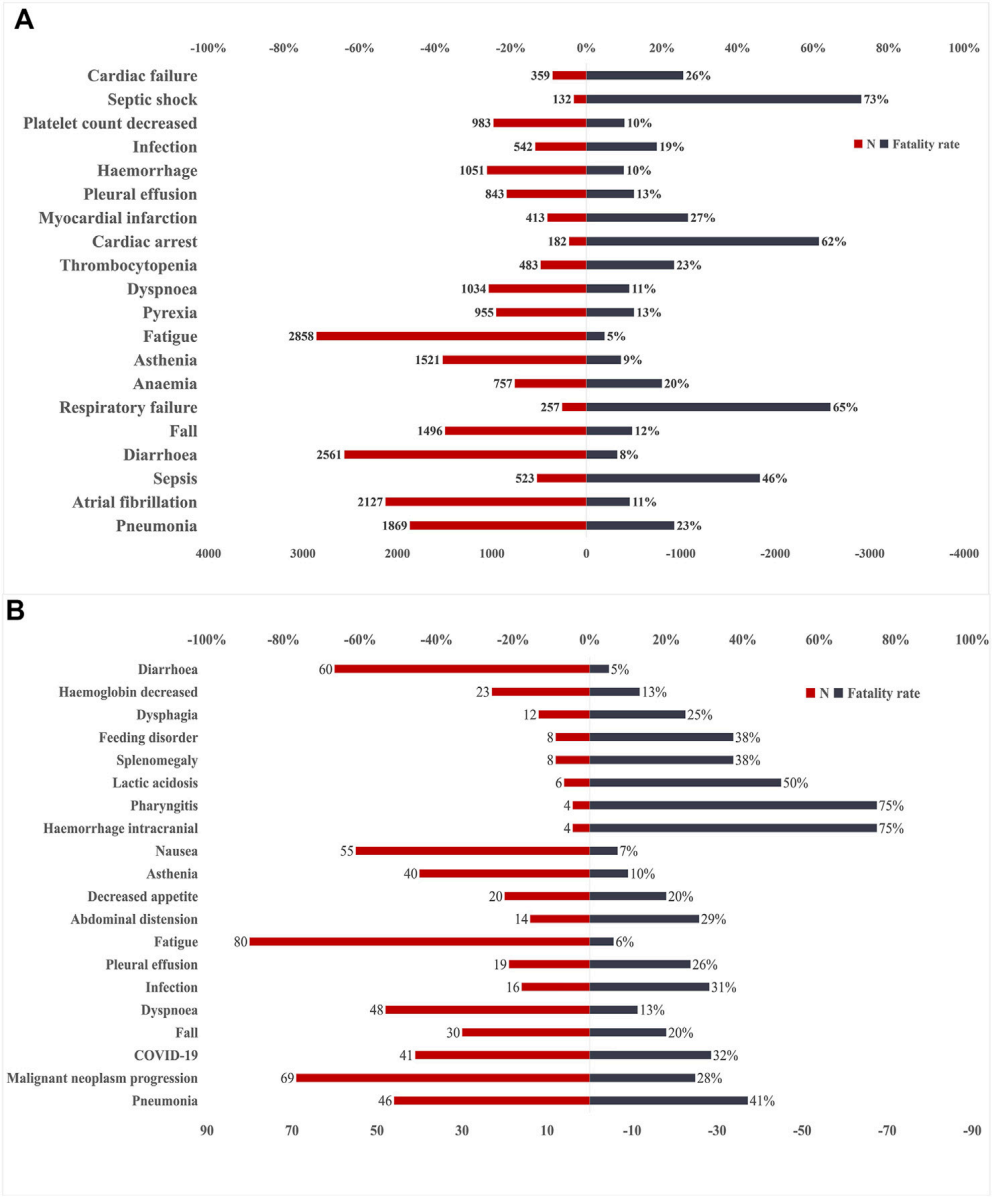
Number of reports and fatality rates for BTK inhibitor-associated AEs according to death reports. **(A)**Number of reports and fatality rates for ibrutinib-associated AEs. **(B)** Number of reports and fatality rates for acalabrutinib-associated AEs. AEs, adverse effects; BTK, Bruton’s tyrosine kinase; N, indicates the number of reports of BTK inhibitor-associated AEs.

**FIGURE 3:**
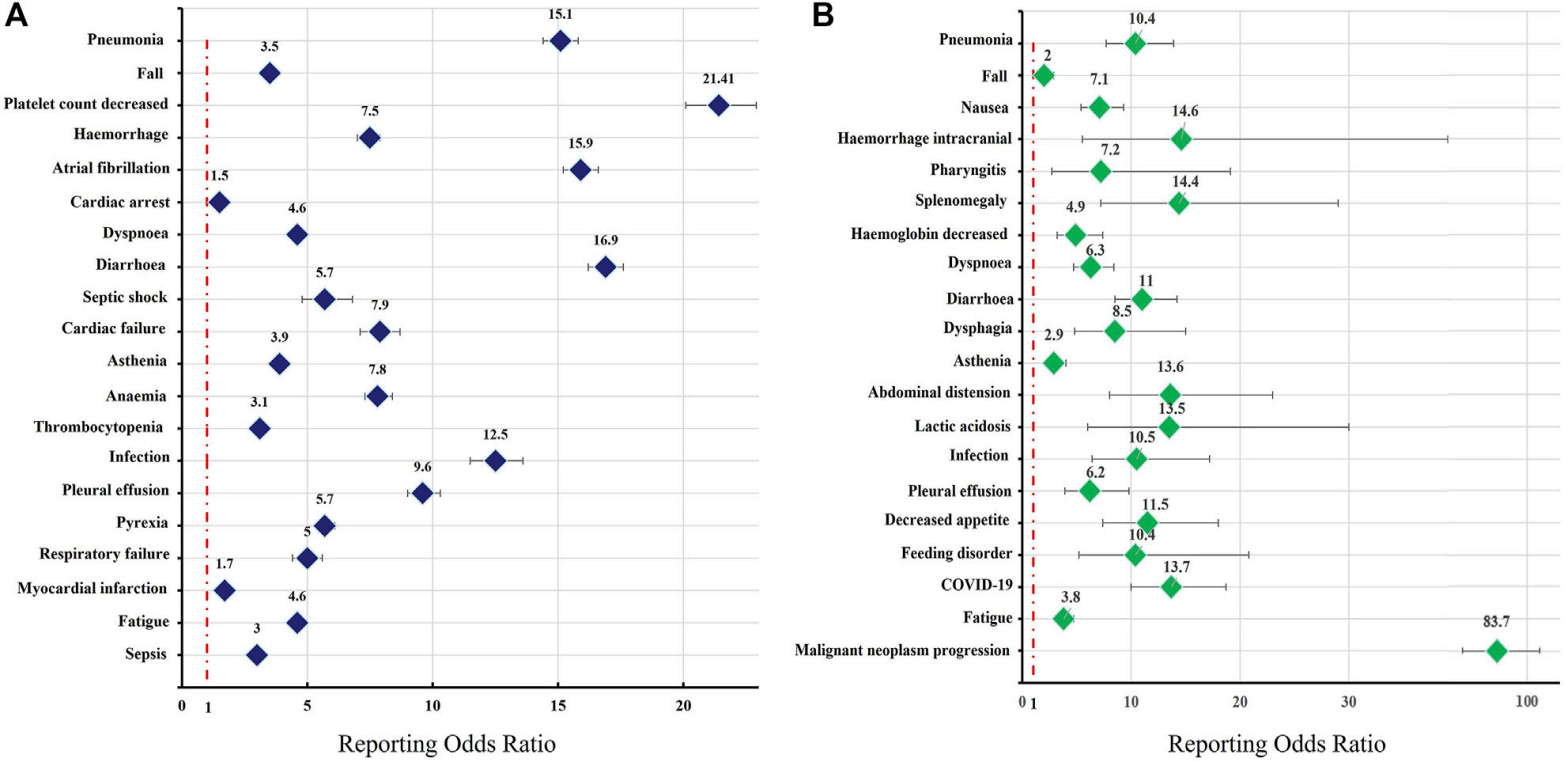
Reporting odds ratios (RORs) of the top 20 AEs associated with BTK inhibitors according to death reports. The graph presents the RORs of BTK inhibitor-associated AEs compared to reports in the full database. Colours represent different drugs: **(A)** ibrutinib; **(B)** acalabrutinib. Error bars represent the 95% confidence interval (CI). A lower limit of the ROR 95% CI above 1 was considered significant. AEs, adverse effects; BTK, Bruton’s tyrosine kinase.

**FIGURE 4:**
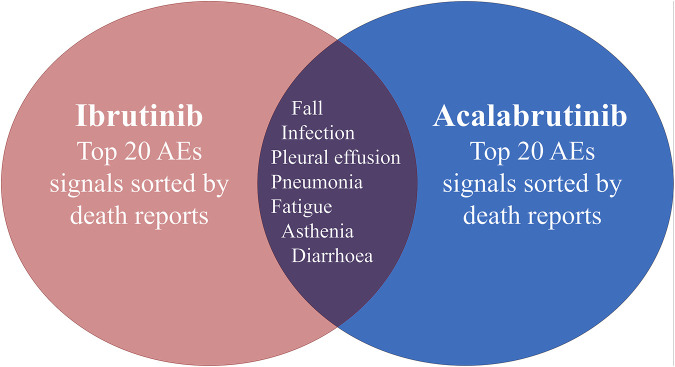
Common preferred terms (PTs) for both ibrutinib and acalabrutinib according to death reports.

The safety issues of BTK inhibitors have hindered their clinical application, but BTK inhibitors could change the era of BCM treatment; thus, we further analysed the potential fatality rates associated with BTK inhibitor regimens. Figure 2 shows the fatality rates and the number of reports, and Supplementary Figure S1 shows the SOC distribution of the top 20 AEs associated with BTK inhibitors according to the number of death reports. Figure 3 and Supplementary Table S4 show the signal strength according to ROR. In general, this frequency was much higher for ibrutinib than for acalabrutinib, which could be due to the shorter marketing time and improved safety of acalabrutinib. In addition to infections, cardiac disorders were an important death cause with ibrutinib, whereas neoplasms benign, malignant, and unspecified was an important cause of death with acalabrutinib. It was observed that the incidences of pneumonia (434, 19, respectively), pleural effusion (106, 5, respectively), a fall (181, 6, respectively), infection (101, 5, respectively), asthenia (139, 4, respectively), diarrhoea (209, 3, respectively), and fatigue (137, 5, respectively) were relatively higher with both ibrutinib and acalabrutinib (Figure 4). We further analysed the population characteristics in Table 4 and found that the deaths caused by pneumonia, pleural effusion, a fall, infection, asthenia, diarrhoea, and fatigue more often occurred in males and elderly people.

**TABLE 4:** Details concerning patients with deaths associated with ibrutinib and acalabrutinib -related AEs in the FAERS database (January 2004 to Dem 2021).

	Pneumonia	Pleural effusion	Fall	Infection	Asthenia	Diarrhoea	Fatigue
Gender							
Femle	119(27.4) /3(15.8)	31(2.8)/2(40)	64(35.4)/1(16.7)	16(15.8)/0	43(30.9)/0	54(25.8)/0	43(31.4)/1(20)
Male	296(68.2)/16(84.2)	72(29.3)/3(60)	113(62.4)/5(83.3)	43(42.6)/5(100)	91(65.5)/4(100)	104(49.8)/3(100)	82(59.8)/(80)
Unknown or missing	19(4.4)/0	3(67.9)/0	4(2.2)/0	42(41.6)/0	5(3.6)/0	51(24.4)/0	12(8.8)/0
Age(year)							
0-18	2(0.5)/0	/	/	/	/	/	/
18-64	63(14.5)/4(21.0)	13(12.3)/1(20)	12(6.6)/0	10(9.9)/0	14(10.1)/0	29(13.9)/0	16(11.7)/0
≥65	274(63.1)/11(58.0)	64(60.4)/4(80)	128(70.7)/5(83.3)	34(33.7)/5(100)	98(70.5)/3(75)	110(52.6)/3(100)	87(63.5)/5(100)
Unknown or missing	95(21.9)/4(21.0)	29(27.3)/0	41(22.7)/1(16.7)	57(56.4)/0	27(29.4)/1(25)	70(33.5)/0	34(24.8)/0
Reporter							
Medical staff	284(65.5)/7(36.8)	46(43.4)/2(40)	98(54.1)/2(33.3)	71(70.3)/3(60)	72(51.8)/0	148(70.8)/0	60(43.8)/1(20)
Non-medical staff	149(34.3)/7(36.8)	60(56.6)/1(20)	83(45.9)/(33.3)	30(29.7)/1(20)	67(48.2)/2(50)	60(28.7)/2(66.7)	77(56.2)/1(20)
Unknown or missing	1(0.2)/5(26.2)	0/2(40)	0/2(33.3)	0/1(20)	0/2(50)	1(0.5)/1(33.3)	0/3(60)
Reporting time							
2014	28(6.5)/0	7(6.6)/0	6(3.3)/0	2(2.0)/0	9(6.5)/0	16(7.7)	6(4.4)/0
2015	56(12.9)/0	17(16.0)/0	11(6.1)/0	6(5.9)/0	23(16.5)/0	22(10.5)	14(10.2)/0
2016	54(12.4)/0	14(13.2)/0	20(11.0)/0	6(5.9)/0	17(12.3)/0	28(13.4)	23(16.8)/0
2017	47(10.8)/0	13(12.3)/0	28(15.5)/0	15(14.8)/0	25(18.0)/0	33(15.8)	25(18.2)/0
2018	59(13.6)/3(15.8)	22(20.7)/1(20)	39(21.5)/0	18(17.8)/0	12(8.6)/0	21(10.0)	15(10.9)/0
2019	71(16.4)/1(5.3)	14(13.2)/0	26(14.4)/1(16.7)	24(23.9)/0	12(8.6)/0	30(14.4)/1(16.7)	20(14.6)/1(20)
2020	66(15.2)/6(31.6)	13(12.3)/2(40)	32(17.7)/2(33.3)	11(10.9)/5(100)	25(18.0)/0	44(21.0)	17(12.4)/0
2021	53(12.2)/9(44.3)	6(5.7)/(40)	19(10.5)/3(50.0)	19(18.8)/0	16(11.5)/4(100)	15(7.2)/2(66.7)	17(12.4)/4(80)

Values are n (%), unless otherwise indicated.ibrutinib vs. acalabrutinib.

### Occurrence time of ibrutinib-related adverse events

**FIGURE 5:**
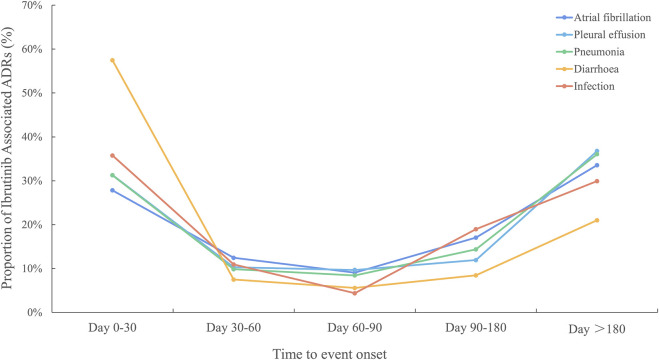
Time to event onset caused by infections, pneumonia, pleural effusion, atrial fibrillation, and diarrhoea following BTK inhibitor regimens. BTK, Bruton’s tyrosine kinase.

As ibrutinib was associated with high frequencies of both AEs and related deaths, we analysed the time to onset for ibrutinib-related AEs. Pneumonia, pleural effusion, atrial fibrillation, diarrhoea, and infection were the top 5 PTs, ordered by signal strength, which were important causes of death, excluding investigations (Supplementary Table S4). The occurrence times of ibrutinib-related AEs are shown in Figure 5. From the data, the AEs diarrhoea and infection both occurred mainly in the first 30 days after the first dose of ibrutinib. In contrast, pneumonia, pleural effusion, and atrial fibrillation mainly occurred after a longer period of ibrutinib use (>180 days).

## Discussion

Due to the limited pre-clinical data, it was under experts’ opinion that pharmacovigilance data mining from the post-marketing adverse event reporting system could provide useful supplements to the drug instruction. Targeted therapy in hematology got a rapid development in recent years. When it comes to lymphocytic malignancy, the application of BTK inhibitors was a milestone especially in the fields of chronic lymphoma leukemia (CLL) therapy as well as graft versus host diseases after allogeneic hematopoietic stem cell transplantation. However, the safety issues hindered its application and affected the prognostic outcome of the patients. Based on the above concerns, our study focused on analyzing and comparing the association of patients prognosis and AEs induced by BTK inhibitors based on the FAERS pharmacovigilance database in the real-world practice, in order to provide novel perspective in lymphocytic malignancies treatments.

According to the results, there were 13 and 12 novel AEs signals were not included in drug labels of ibrutinib and acalabrutinib, respectively. It was estimated that approximately 40% of patients treated with ibrutinib suspend targeted therapy owing to serious intolerances (some even fatal), which suggests that more attention should be paid to BTK inhibitor safety issues.

Due to their structural differences, the potential AE signals and types vary for ibrutinib and acalabrutinib; further, the AE frequencies for ibrutinib seemed to be higher than those for acalabrutinib. This may result from the higher bruton’s tyrosine kinase (BTK) selectivity and target specificity of acalabrutinib, thus reducing the occurrence of off-target effects (Byrd et al., 2016; Wen et al., 2021). Studies have found the AEs of ibrutinib mainly due to its blocks of BTK activity, *via* the irreversible binding to Cys481 in the kinase domain and also covalent or non-covalent binding to other homologous kinases regardless of cysteine residues, including TEC family kinases (TEC, ITK, RLK, BMX), epidermal growth factor receptor (EGFR), ERBB2/HER2 human epidermal growth factor receptor 2, ERBB4/HER4 human epidermal growth factor receptor, B-lymphoid kinase (BLK), and Janus kinase 3 (JAK3), thereby resulting in off-target effects (Paydas 2019; Liu et al., 2021). When it comes to acalabrutinib was shown to have 323-, 94-,19-, and 9-fold higher selectivity for the TEC family kinases ITK, TXK, BMX, and TEC, respectively, compared to that of ibrutinib, and shows no activity toward EGFR. Moreover, compared with ibrutinib, acalabrutinib shows almost no inhibitory effect on the activity of ITK, EGFR, ERBB2, ERBB4, JAK3, LYN, LCK, SRS, and YES1, even at a higher half-inhibitory concentration (>1000 nM) (Wu, Zhang, and Liu 2016).

Moreover, some AEs of BTKs inhibitors were fatal, which was also a critical concerns in clinical practice. When concerning the mortality related to AEs, infection, pneumonia, haemorrhage, pleural effusion, and diarrhoea ranked in the top 20 for both ibrutinib and acalabrutinib according to the FARES database death reports. Infection, pleural effusion, and pneumonia may result from the inherent humoral immunosuppression of haematological diseases and treatment-related immunosuppression, which were also the vital reasons underlying the morbidity and mortality associated with BCM. Studies indicated that 4.1% of patients treated with ibrutinib suffer from opportunistic infections, and among them, invasive fungal infections (IFIs) account for approximately half the AE reports, indicating that ibrutinib might be related to early IFIs and likely induce myelosuppression (Ghez et al., 2018). This result was consistent with our results indicating that the ROR of fungal infection was relatively stronger for infectious pneumonia with ibrutinib. The mechanism of IFIs were complex and the involvement of detrimental effects on phagocytes was on of the important factors, possibly caused by neutropenia induced during the progression of malignancies as well as the off-target effects of BTK inhibitors on intracellular signalling components (Estupinan and Berglof et al., 2021). Besides, haemorrhage was also a common AE associated with BTK inhibitors, which mainly caused by inhibition of the TEC family kinases BTK and TEC and the fatality was also ranked high in our study. Researchers have found BTK and TEC could regulate platelet activation through collagen receptor glycoprotein VI (GPVI) *via* the phosphorylation of phospholipase Cγ2 (PLCγ2), and with arterial shear, the interaction between platelets and the damaged vessel wall is mainly mediated by the combination of von Willebrand factor (VWF) and its receptor GPIb-IXV complex (Quek, Bolen, and Watson 1998; Atkinson, Ellmeier, and Watson 2003). Diarrhoea was another AE found to be associated with the use of BTK inhibitors, which is also often related to inhibition of the EGFR. However, studies have found that the EGFR off-target effect alone is not sufficient to explain the mechanism underlying diarrhoea in patients treated with BTK inhibitors, which suggests that mechanisms other than binding to kinases harbouring a cysteine in the active site contribute to diarrhoea (Byrd et al., 2016; Estupinan and Berglof et al., 2021). Further, another fatal AE signal, atrial fibrillation (AF), was reported to be associated with a higher mortality rate and was considered one of the main reasons for patient intolerance (Salem et al., 2019). Our study found that the incidence of atrial fibrillation-related fatality was relatively higher in ibrutinib after its long-term time use; the underlying pathogenetic mechanism may be due to its inhibitory effect on multiple kinases, including ERBB2 and the PI3K-AKT pathway, resulting in the off-target effects of ibrutinib at its therapeutic concentration (McMullen et al., 2014; Tenin et al., 2014). Besides, it was found that mutations in the *Erbb2* gene could impaire atrial electrical signalling; when PI3K activity is decreased, the susceptibility to atrial fibrillation was found to be increased in mouse model. Moreover, ibrutinib also acts specifically on atrial myocytes and shorten the action potential time course of atrial myocytes, thereby shortening the non-return period and increasing the risk of atrial fibrillation, which was fatal especially in some elderly patients (Jiang and Li et al., 2019).

Based on its efficacy of BCM treatment, the development of novel BTK inhibitors was under rapid increase. Zanubrutinib and Orelabrutinib were the latest approved BTK inhibitors by CFDA, which were a new generation of BTK inhibitors marked with higher target selectivity and thus less safety concerns. However, due to the short marketing time as well as the limited market access, it was impossible to make a credible pharmacovigilance study at present.

According to the public data available, zanubrutinib has a higher target occupancy rate and exhibits less off-target binding than ibrutinib, and clinical trials indicated that it is generally well-tolerated in patients (Tam, Quach, et al., 2020; Tam, Robak, et al., 2020; Xu et al., 2020). Besides, orelabrutinib has high selectivity, irreversible binding ability to BTK, and little activity on other kinases (ITK, EGFR, ERBB2, etc.). Studies have shown that at a dose of 50 mg or higher dose, orelabrutinib could almost completely bind to BTK, with a binding rate greater than 99% and low inter-variability (Dhillon 2021). Meanwhile, the off-target effects of orelabrutinib were minimized even in a long-time use after researchers’ years dedication (Song et al., 2020).

However, although data mining techniques have advantages in analysing clinical safety issues in real-world, there were also certain limitations. Firstly, the FAERS database is a spontaneous reporting system (SRS), and the data mining technology that was used in this study could not improve its inherent limitations, such as false reporting, uneven information quality, under-reporting, and inaccuracy, all of which might result in reporting bias. Secondly, the SRS, which is used in qualitative research, cannot be used to quantify adverse reaction signals based on the total number of adverse reactions, which made it impossible to calculate the incidence of each adverse reaction. Thirdly, although data mining techniques could provide a profile of BTK inhibitor-associated adverse reaction signals through signal detection, this is insufficient to prove a causal relationship. Last but not least, due to their short marketing time, our study only analyzed and compared the safety signal of ibrutinib and acalabrutinib, future studies would be made to design a larger pharmacovigilance research. Above all, though the apparent defects and limits, real-world data mining and pharmacovigilance study could provide rational considerations for physicians to choose the suitable BTK inhibitors for different patients to obtain desired tolerance and efficacy.

Consequently, our pharmacovigilance analysis of the FAERS database identified various novel AE signals for BTK inhibitors in a real-world practice setting. We made a comprehensively study and compared the AEs signals of iburintib and acalabrutinib, our results indicated that patients taking ibrutinib are more prone to induce AEs than those taking acalabrutinib. When it comes to fatality, it was found that infection, pneumonia, pleural effusion, and diarrhoea were prevalent both in patients’ taking ibrutinib and acalabrutinib, especially in elderly patients. Furthermore, atrial fibrillation was a fatal AE associated with ibrutinib, which exhibited strikingly higher death rates. Our results indicated that more attention should be paid to cardiovascular toxicities with ibrutinib, which suggest that more careful pharmaco-monitoring, such as precise pharmacokinetic analysis and therapeutic drug monitoring, could be implemented in the future in order to provide a more sustainalbe and safety therapy in lymphocytic maligancies.

## Data Availability

The original contributions presented in the study are included in the article/Supplementary Material, further inquiries can be directed to the corresponding authors.

## Abbreviations

BTK, Bruton’s tyrosine kinase; FDA, the US Food and Drug Administration; FAERS, the FDA Adverse Event Reporting System; BCM, B-cell malignancy; SRS, spontaneous reporting system; ROR, reported odds ratio method; PRR, proportional report ratio method; PT, preferred term; AE, adverse event; MedDRA, Medical Dictionary for Regulatory Activities; SOC, system organ class; IFI: invasive fungal infection; MCL, mantle cell lymphoma; MZL, marginal zone lymphoma; WM, Waldenstrom macroglobulinemia; CLL, chronic lymphocytic leukaemia.
