# Supplementary material for: Data mining and safety analysis of BTK inhibitors: A pharmacovigilance investigation based on the FAERS database
Source: Front Pharmacol. 2022 Nov 11;13:995522. doi: 10.3389/fphar.2022.995522 (PMC9691840; doi:10.3389/fphar.2022.995522)
Supplement: Supplementary file 1 [file DataSheet1.ZIP › Supplementary materials/Supplementary materials l.docx]

| Supplementary Table S1 Calculation formulas and judgment criteria of two risk signal analysis methods | | |  |
| --- | --- | --- | --- |
| Method | Calculation formulas | Judgement criteria | |
| ROR | ROR=ad/bc | 95%CI>1，N≥3 | |
|  | 95%CI=eln(ROR)±1.96(1/a+1/b+1/c+1/d)^0.5 |  |  |
| PRR | PRR=a(c+d)/c(a+b) | PRR≥2，χ^2^≥4，N≥3 | |
|  | χ^2^=［(ad-bc)^2］(a+b+c+d)/［(a+b)(c+d)(a+c)(b+d)］ |  |  |
| BCPNN | IC = log_2_a(a + b + c + d)(a + c)(a + b) | IC025> 0 | |
|  | 95% CI = e^ln(IC) ± 1.96(1/a+1/b+1/c+1/d)^0.5^ |  | |
| MGPS | EBGM = a(a + b + c + d)/(a + c)/(a + b) | EBGM05> 2, | |
|  | 95% CI = e^ln(EBGM) ± 1.96(1/a+1/b+1/c+1/d)^0.5^ | N>0 | |
| Note：ROR, Reporting Odds Ratio; PRR, Proportional Reporting Ratio; a, the number of reports with suspect adverse drug event (ADE) of the suspect drug;b: the number of reports with the suspect ADE of all other drugs; c: the number of reports with all other ADEs of the suspect drug; d: the number of reports with all other ADEs of all other drugs;CI: confidence interval; N: the number of co-occurrences;χ2:chi-squared; BCPNN: Bayesian confidence propagation neural network; IC: information component; IC025: the lower limit of the 95% two-sided CI of the IC; MGPS: multi-item gamma Poisson shrinker; EBGM:empirical Bayesian geometric mean; EBGM05: the lower 95% one-sided CI of EBGM. | | |  |

Supplementary Table S2 The signal strength of SOC level

|  | Acalabrutinib | | | | | | Ibrutinib | | | | |
| --- | --- | --- | --- | --- | --- | --- | --- | --- | --- | --- | --- |
| SOC | N | ROR | PRR | | IC | EBGM | N | ROR | PRR | IC | EBGM |
|  |  | (95% two-sided CI) | (χ2) | | (95% two-sided CI) | (95% one-sided CI) |  | (95% two-sided CI) | (χ2) | (95% two-sided CI) | (95% one-sided CI) |
| Metabolism and nutrition disorders | 96 | 1.2 (0.97,1.47) | 1.19 (2.96) | 60.50 (49.19,74.40) | | 2569744.48 (2089431.25) | 2594 | 1.09 (1.14,18.82) | 1.09 (18.82) | 70.13 (67.40,72.98) | 2048616658.79 (1968778976.38) |
| Ear and labyrinth disorders | 11 | 0.83 (0.46,1,49) | 0.83 (0.4) | | 54.77 （30.26,99.11） | 1790799.10 （989509.60） | 622 | 1.60 (1.47,1.73) | 1.59 (135.56) | 65.47 (60.48,70.88) | 2987559652.77 (2759670360.66) |
| Hepatobiliary disorders | 3 | 0.08 (0.02,0.23) | 0.08 (33.87) | | 54.43 （17.54,168.97） | 167766.95 （54044.61） | 542 | 0.47 (0.43,0.51) | 0.47 (325.65) | 66.81 (61.38,72.72) | 894244884.66 (821555016.08) |
| Infections and infestations | 157 | 0.87 (0.74,1.02) | 0.88 (2.83) | | 62.34 （52.83，73.56） | 1910633.30 （1619192.37） | 9720 | 2.10 (2.05,2.15) | 1.86 (4334.23) | 73.18 (71.54,74.85) | 3489924036.48 (3411865337.63) |
| Investigations | 275 | 1.44 (1.26,1.64) | 1.36 (29.99) | | 63.34 (55.56,72.21) | 2940806.01 （2579529.96） | 10363 | 1.97 (1.93,2.01) | 1.74 (3741.21) | 73.46 (71.85,75.10) | 3269574221.08 (3198057032.74) |
| Nervous system disorders | 229 | 0.80 (0.7,0.92) | 0.83 (9.4) | | 63.51 (55.16,73.13) | 1805374.48 （1568021.39） | 7347 | 0.89 (0.86,0.91) | 0.91 (88.26) | 73.40 (71.58,75.27) | 1708889089.99 (1666493550.26) |
| Injury, poisoning and procedural complications | 96 | 0.38 (0.31,0.46) | 0.42 (92.35) | | 62.00 (50.41,76.25) | 905103.09 （735934.97） | 6493 | 0.95 (0.93,0.98) | 0.96 (14.49) | 72.96 (71.06,74.92) | 1806110172.59 (1759028733.82) |
| Musculoskeletal and connective tissue disorders | 138 | 0.89 (0.75,1.06) | 0.90 (1.64) | | 61.94 (51.98,73.80) | 1954713.50 （1640451.62） | 6880 | 1.63 (1.58,1.67) | 1.53 (1389.32) | 72.46 (70.61,74.35) | 2875176326.20 (2801907242.57) |
| Congenital, familial and genetic disorders | 3 | 0.22 (0.07,0.67) | 0.22 (8.57) | | 52.95 (17.06,164.36) | 469643.60 （151290.24） | / | / | / | / | / |
| Respiratory, thoracic and mediastinal disorders | 147 | 0.93 (0.78,1.1) | 0.94 (0.74) | | 62.07 (52.34,73.6) | 2026354.02 （1708883.52） | 7287 | 1.69 (1,65.1,73) | 1.57 (1699.68) | 72.58 (70.77.74.44) | 2963594480.00 (2889774300.41) |
| Psychiatric disorders | 46 | 0.22 (0.17.0.3) | 0.25 (121.41) | | 60.64 (45.21,81.33) | 533945.24 （398090.31) | 1726 | 0.28 (0.27,0.30) | 0.31 (2982.37) | 70.75 (67.43,74.24) | 591087394.58 (563290214.01) |
| Neoplasms benign, malignant and unspecified (incl cysts and polyps) | 92 | 0.84 (0.68,1.04) | 0.85 (2.59) | | 60.85 (49.27,75.15) | 1843801.38 (1492980.49) | 1603 | 0.48 (0.46,0.51) | 0.50 (854.50) | 69.86 (66.46,73.43) | 947833052.59 (901676504.73) |
| Immune system disorders | 19 | 0.65 (0.41,1.02) | 0.65 (3.56) | | 56.68 (36.05,89.13) | 1416432.08 (900811.47) | 570 | 0.66 (0.61,0.72) | 0.66 (98.64) | 66.47 (61.19,72.20) | 1253686727.07 (1154152108.99) |
| Endocrine disorders | / | / | / | | / | / | 118 | 0.42 (0.35,0.5) | 0.42 (93.84) | 62.58 (52.23,74.98) | 797527231.79 (665622367.76) |
| Skin and subcutaneous tissue disorders | 118 | 1.01 (0.84,1.22) | 1.01 (0.02) | | 61.32 (50.80,74.01) | 2190887.35 (1815146.69) | 6917 | 2.21 (2.15,2.26) | 2.02 (3818.25) | 72.08 (70.24,73.96) | 3789028175.80 (3692620369.09) |
| General disorders and administration site conditions | 845 | 3.40 (3.07,3.77) | 2.02 (610.05) | | 66.00 (59.52,73.19) | 4382477.98 (3952296.03) | 16615 | 1.57 (1.54,1.60) | 1.35 (2090.06) | 75.18 (73.74,76.65) | 2542350353.06 (2493540574.95) |
| Renal and urinary disorders | 40 | 0.49 (0.36,0.67) | 0.51 (20.49) | | 59.20 (43.24,81.06) | 1094641.66 (799488.68) | 1852 | 0.78 (0.82,106.31) | 0.79 (69.62) | 69.62 (66.45,72.94) | 1495288465.83 (1427201039.96) |
| Reproductive system and breast disorders | 3 | 0.14 (0.04,0.43) | 0.14 （15.93） | | 53.57 (17.26,166.29) | 305481.38 (98407.71) | 388 | 0.61 (0.56,0.68) | 0.62 (93.22) | 65.47 (59.23,72.35) | 1165647510.98 (1054674230.13) |
| Gastrointestinal disorders | 250 | 1.02 （0.89,1,17） | 1.01 （0.07） | | 63.48 (55.41,72.73) | 2199132.22 (1919383.43) | 11910 | 1.88 (1.85,1.92) | 1.64 (3572.67) | 73.94 (72.39,75.52) | 3090972299.97 (3026354379.59) |
| Cardiac disorders | 85 | 0.71 （0.57,0.89） | 0.73 （9.23） | | 60.85 (48.88,75.74) | 1581434.82 (1270378.72) | 6347 | 2.00 (1.95,2.05) | 1.85 (2868.12) | 71.95 (70.06,73.90) | 3483957824.35 (3392190221.62) |
| Vascular disorders | 42 | 0.52 （0.38,0.71） | 0.53 （18.11） | | 59.26 (43.60,80.55) | 1155864.60 (850438.07) | 3410 | 1.51 (1.46,1.56) | 1.47 (539.13) | 70.49 (68.06,73.00) | 2768752094.04 (2673413907.64) |
| Blood and lymphatic system disorders | 165 | 2.61 （2.22,3.07） | 2.43 （145.77） | | 61.02 (51.90,71.75) | 5268202.25 (4480521.49) | 4271 | 2.26 (2.19,2.34) | 2.14 (2703.24) | 70.60 (68.40,72.87) | 4023278402.54 (3897817395.61) |
| Eye disorders | 8 | 0.14 (0.07,0.28) | 0.14 （42.77） | | 56.38 (28.14,112.95) | 309563.18 (154516.60) | 2353 | 1.45 (1.39,1.51) | 1.43 (310.08) | 69.46 (66.63,72.42) | 2686292206.74 (2576745410.62) |
| Abbreviations: N: the number of reports of BTK-associated AEs; ROR: reporting odds ratio; CI: confidence interval; PRR: proportional reporting ratio; χ2 : chi-squared; BTK：Bruton's tyrosine kinase inhibitor; FAERS:the FDA's Adverse Event Reporting System; | | | | | | | | | | | |

Supplementary Table S3. The signal strength of PT at Infections and infestations, Cardiac disorders, Blood lymphatic system disorders and Vascular disorders of the two BTK inhibitors

| ibrutinib | | | | | | | acalabrutinib | | | | | |
| --- | --- | --- | --- | --- | --- | --- | --- | --- | --- | --- | --- | --- |
| SOC | PT | N | ROR | PRR | IC | EBMG | PT | N | ROR | PRR | IC | EBGM |
|  |  |  | (95% two-sided CI) | (χ2) | (95% two-sided CI) | (95% one-sided CI) |  |  | (95% two-sided CI) | (χ2) | (95% two-sided CI) | (95% one-sided CI) |
| Infections and infestations | Cerebral aspergillosis | 24 | 150.4 (92.9, 243.7) | 150.4 (2451) | 50 (30.9, 81.1) | 195790389918.9 (120870358877.4) | Fungal infection | 7 | 23.5 (11.2, 49.4) | 23.4 (149.7) | 48.6 (23.1, 102.3) | 50572769.7 (24045688.2) |
|  | Disseminated cryptococcosis | 10 | 94.9 (47, 191.6) | 94.9 (722.4) | 48 (23.8, 96.9) | 139591296516.2 (69121186260.3) | Clostridium difficile colitis | 4 | 21.2 (7.9, 56.6) | 21.1 (76.6) | 47.2 (17.7, 126) | 45689133.2 (17106770.8) |
|  | Nasal abscess | 4 | 94.9 (31.2, 288.3) | 94.9 (289) | 45.4 (14.9, 137.8) | 139591296516.2 (45945842555.3) | Kidney infection | 3 | 14.5 (4.7, 45) | 14.5 (37.5) | 46.9 (15.1, 145.7) | 31279827.7 (10068186) |
|  | Dermatitis infected | 10 | 89.8 (44.6, 180.5) | 89.7 (690.8) | 48.1 (23.9, 96.7) | 133651241345.3 (66460616026.1) | COVID-19 | 41 | 13.7 (10, 18.7) | 13.3 (468.7) | 54.6 (40, 74.4) | 28888922.7 (21174510.8) |
|  | Eye infection viral | 4 | 88.6 (29.4, 266.8) | 88.5 (273.3) | 45.4 (15.1, 136.9) | 132244386173.3 (43888739645) | Nasopharyngit-is | 7 | 11.1 (5.3, 23.3) | 11 (63.9) | 49.7 (23.7, 104.5) | 23914947.4 (11376046.7) |
|  | Pneumonia cryptococcal | 18 | 86.7 (51.6, 145.6) | 86.6 (1208.2) | 49.8 (29.6, 83.7) | 129964310549.6 (77356775400.5) | Infection | 16 | 10.5 (6.4, 17.2) | 10.4 (136.2) | 52.2 (31.9, 85.4) | 22548198.1 (13773021) |
|  | Cerebral fungal infection | 9 | 71.2 (34.6, 146.2) | 71.2 (512.7) | 48 (23.4, 98.7) | 110851911939.4 (53957423727.2) | Cellulitis | 7 | 8.2 (3.9, 17.2) | 8.1 (43.9) | 50.2 (23.9, 105.4) | 17645980.6 (8394890.9) |
|  | Infectious pleural effusion | 6 | 55.3 (23.3, 131.4) | 55.3 (274.4) | 47.2 (19.9, 112) | 89737262046.1 (37809333686.3) | Escherichia infection | 3 | 8.1 (2.6, 25.3) | 8.1 (18.7) | 47.7 (15.4, 148.2) | 17576612.1 (5659529.2) |
|  | Metapneumovirus infection | 5 | 53.6 (20.8, 137.8) | 53.6 (222.1) | 46.7 (18.2, 120.1) | 87244560322.6 (33922629008.5) | Aspergillus infection | 3 | 8 (2.6, 25) | 8 (18.4) | 47.7 (15.4, 148.3) | 17371065.6 (5593375.5) |
|  | Meningitis fungal | 3 | 52.4 (15.5, 177.2) | 52.4 (130.7) | 45.2 (13.4, 152.9) | 85658295589.5 (25346954440.5) | Influenza | 3 | 5.5 (1.8, 17) | 5.5 (10.9) | 48.3 (15.6, 150) | 11824029.7 (3807825.5) |
| Cardiac disorders | Ventricular tachyarrhythmia | 8 | 88.6 (40.6, 193.2) | 88.5 (546.7) | 47.4 (21.7, 103.5) | 132244386173.3 (60623872720.7) | Cardiac tamponade | 3 | 11.9 (3.8, 37) | 11.9 (29.9) | 47.2 (15.2, 146.5) | 25713932.4 (8277890.5) |
|  | Cardiac flutter | 54 | 21.9 (16.7, 28.9) | 21.9 (1011.5) | 54.7 (41.5, 72.1) | 38899868180.6 (29531110526.8) | Acute coronary syndrome | 3 | 6.6 (2.1, 20.4) | 6.5 (14.1) | 48 (15.5, 149.2) | 14181484.9 (4566737.4) |
|  | Arrhythmia supraventricular | 21 | 17.2 (11.1, 26.7) | 17.2 (305) | 52.3 (33.7, 81.1) | 30965674931.4 (19967830994.7) | Acute myocardial infarction | 5 | 6 (2.5, 14.4) | 6 (20.7) | 49.6 (20.6, 119.5) | 12955541 (5382892.8) |
|  | Atrial fibrillation | 2127 | 15.9 (15.2, 16.6) | 15.1 (26925.9) | 65.8 (63, 68.8) | 27366161329.8 (26174651006.2) | Palpitations | 10 | 5.9 (3.2, 11) | 5.9 (40.4) | 51.7 (27.7, 96.2) | 12707332.8 (6821449.9) |
|  | Pericarditis | 71 | 13.5(10.7, 17.1) | 13.5 (789.9) | 56.2 (44.3, 71.2) | 24545635243.2 (19357023667.3) | Cardiac disorder | 10 | 5.2 (2.8, 9.6) | 5.1 (33.4) | 51.9 (27.8, 96.6) | 11141358.5 (5980952) |
|  | Atrial flutter | 138 | 11.6 (9.8, 13.8) | 11.6 (1291.3) | 58.3 (49.2, 69.1) | 21194668737.5 (17881261980) | Atrial fibrillation | 24 | 4.9 (3.3, 7.3) | 4.8 (73.1) | 54.5 (36.4, 81.5) | 10466117.7 (6991254.1) |
|  | Heart valve calcification | 3 | 11.5 (3.6, 36.2) | 11.5 (27.7) | 47.3 (15, 149.4) | 20938694477.4 (6623398997.2) | Cardiac failure | 7 | 4.4 (2.1, 9.3) | 4.4 (18.4) | 51 (24.3, 107.3) | 9544537.5 (4541353.1) |
|  | Wolff-Parkinson-White syndrome | 4 | 10.5 (3.9, 28.3) | 10.5 (33.2) | 48.2 (17.8, 130.5) | 19180483490.8 (7088936122.6) | Arrhythmia | 5 | 4 (1.7, 9.7) | 4 (11.3) | 50.2 (20.9, 120.8) | 8688841 (3610440.1) |
|  | Pleuropericarditis | 5 | 10.2 (4.2, 24.8) | 10.2 (40.2) | 48.9 (20.1, 119.1) | 18695262926.3 (7677848545.8) | Angina pectoris | 5 | 3.6 (1.5, 8.6) | 3.6 (9.2) | 50.4 (20.9, 121.2) | 7729302.3 (3211790.1) |
|  | Ventricular arrhythmia | 22 | 9.9 (6.5, 15.2) | 9.9 (171.5) | 53.2 (34.8, 81.3) | 18231580943.4 (11929064886.6) | Tachycardia | 10 | 2.5 (1.3, 4.6) | 2.5 (8.8) | 52.9 (28.4, 98.6) | 5357204.1 (2876118.8) |
| Blood and lymphatic system disorders | Haemorrhagic diathesis | 141 | 93.8 (77.8, 113) | 93.5 (10064.3) | 55.7 (46.2, 67.1) | 137960557070.9 (114420344637.4) | Anaemia macrocytic | 3 | 34.9 (11.2, 108.5) | 34.8 (98.1) | 45.6 (14.7, 141.9) | 75145186.1 (24159080.1) |
|  | Splenic haematoma | 10 | 87.4 (43.5, 175.4) | 87.4 (676) | 48.1 (24, 96.5) | 130866840484 (65203578111.6) | Aplastic anaemia | 4 | 34.3 (12.8, 91.7) | 34.2 (128.6) | 46.5 (17.4, 124.2) | 73927555.8 (27661671.2) |
|  | Splenic haemorrhage | 17 | 78.4 (46.2, 133.1) | 78.4 (1050.9) | 49.8 (29.3, 84.4) | 119985777342.6 (70722979064.4) | Leukocytosis | 10 | 22.5 (12.1, 42) | 22.4 (203.8) | 49.7 (26.7, 92.7) | 48366960.6 (25950513.4) |
|  | Lymphocytosis | 164 | 65.2 (55.2, 77.1) | 65 (8641.4) | 56.5 (47.8, 66.8) | 102812751326.3 (86945867135.9) | Autoimmune haemolytic anaemia | 4 | 19.4 (7.3, 51.8) | 19.3 (69.4) | 47.3 (17.7, 126.3) | 41829343.9 (15662995.3) |
|  | Autoimmune neutropenia | 7 | 54.1 (24.3, 120.2) | 54.1 (313.5) | 47.6 (21.4, 105.9) | 87942516805.2 (39558930835.6) | Haemolytic anaemia | 4 | 16.7 (6.3, 44.6) | 16.7 (58.8) | 47.5 (17.8, 126.9) | 36040856.6 (13497297) |
|  | Increased tendency to bruise | 387 | 52.3 (47, 58.2) | 51.8 (16692.3) | 59.3 (53.2, 66) | 84821438549.5 (76172762918.2) | Agranulocytosis | 3 | 8.6 (2.8, 26.8) | 8.6 (20.2) | 47.6 (15.3, 147.9) | 18654616.9 (6006466.1) |
|  | Leukostasis syndrome | 11 | 51.5 (27.3, 97.1) | 51.4 (471.1) | 49 (26, 92.5) | 84265477775 (44647984850.5) | Febrile neutropenia | 21 | 6.5 (4.2, 10) | 6.4 (96) | 53.7 (34.9, 82.6) | 13879919.8 (9020622.9) |
|  | Lymph node pain | 30 | 50.9 (34.6, 74.7) | 50.8 (1270.8) | 51.9 (35.4, 76.3) | 83384181547.3 (56774441613.9) | Cytopenia | 3 | 5.7 (1.8, 17.8) | 5.7 (11.7) | 48.2 (15.5, 149.8) | 12373771.4 (3984806.9) |
|  | Retroperitoneal lymphadenopathy | 6 | 39.9 (17.1, 92.9) | 39.8 (202.9) | 47.6 (20.4, 111) | 67302946534.6 (28856487600.1) | Anaemia | 19 | 5.6 (3.6, 8.9) | 5.6 (71.6) | 53.6 (34.1, 84.3) | 12091451 (7688965.5) |
|  | Spleen atrophy | 3 | 38.3 (11.6, 126.6) | 38.3 (97.7) | 45.6 (13.8, 150.8) | 64982155274.8 (19667467664.9) | Bone marrow failure | 4 | 2.9 (1.1, 7.7) | 2.9 (4.9) | 50 (18.8, 133.5) | 6243734.9 (2339882.3) |
| Vascular disorders | Capillary fragility | 12 | 159.4 (80.1, 317.3) | 159.4 (1276.1) | 48 (24.1, 95.5) | 203727838158.8 (102349265405.1) | Orthostatic hypotension | 4 | 11 (4.1, 29.4) | 11 (36.3) | 48.1 (18, 128.4) | 23815588.4 (8921447) |
|  | Vascular rupture | 43 | 70.1 (50.4, 97.4) | 70 (2415.2) | 52.6 (37.8, 73) | 109355934720.2 (78694996142) | Haematoma | 6 | 4.3 (1.9, 9.7) | 4.3 (15.4) | 50.6 (22.7, 112.9) | 9386936.3 (4209484.9) |
|  | Lymphoedema | 53 | 22.3 (16.9, 29.5) | 22.3 (1009.4) | 54.6 (41.4, 72.2) | 39492911292 (29899758400.7) | Haemorrhage | 15 | 3 (1.8, 5.1) | 3 (20.3) | 53.8 (32.3, 89.5) | 6544272.5 (3934738.4) |
|  | Aortic intramural haematoma | 3 | 11.9 (3.7, 37.5) | 11.9 (28.8) | 47.2 (14.9, 149.4) | 21660718424.9 (6847101129.9) | Hypotension | 17 | 1.9 (1.2, 3.1) | 1.9 (7.3) | 54.8 (34, 88.4) | 4119379.9 (2553634.9) |
|  | Aortic aneurysm | 41 | 10.4 (7.6, 14.1) | 10.3 (335.7) | 55 (40.3, 75) | 18979067212.4 (13906572074.8) | / |  | / | / | / | / |
|  | Poor peripheral circulation | 20 | 8.4 (5.4, 13.1) | 8.4 (127.7) | 53.2 (34.1, 82.9) | 15548535503 (9974882594.1) | / |  | / | / | / | / |
|  | Haematoma | 320 | 8 (7.2, 9) | 8 (1914.4) | 61.2 (54.8, 68.5) | 14770480342.7 (13214961242.3) | / |  | / | / | / | / |
|  | Hypertensive urgency | 4 | 7.9 (2.9, 21.3) | 7.9 (23.6) | 48.6 (18, 131.1) | 14608391495.9 (5419227870.9) | / |  | / | / | / | / |
|  | Bloody discharge | 9 | 7.8 (4, 15.2) | 7.8 (52.3) | 51 (26.3, 98.7) | 14458945035.6 (7465267533.6) | / |  | / | / | / | / |
|  | Haemorrhage | 1051 | 7.5 (7, 7.9) | 7.3 (5618.9) | 64.8 (60.9, 68.9) | 13528350584.5 (12716856085.1) | / |  | / | / | / | / |
|  | Abbreviations: N: the number of reports of BTK-associated AEs; ROR: reporting odds ratio; CI: confidence interval; PRR: proportional reporting ratio; χ2 : chi-squared; BTK：Bruton's tyrosine kinase inhibitor; FAERS:the FDA's Adverse Event Reporting System; | | | | | | | | | | | |

Supplementary Table S4. The signal strength of the top 20 AEs of BTK inhibitors according to death reports

|  | ibrutinib | | | | | acalabrutinib | | | | |  |
| --- | --- | --- | --- | --- | --- | --- | --- | --- | --- | --- | --- |
| PT | N | ROR | PRR | IC | EBGM | PT | N | ROR | PRR | IC | EBGM |
|  |  | (95% two-sided CI) | (χ2) | (95% two-sided CI) | (95% one-sided CI) |  |  | (95% two-sided CI) | (χ2) | (95% two-sided CI) | (95% one-sided CI) |
| Platelet count decreased | 99 | 21.41 (20.1, 22.9) | 20.95 (17589.3) | 63.1 (59.2, 67.4) | 37285314904.9 (34933376272.7) | Malignant -neoplasm progression | 19 | 83.7 (65.6, 106.6) | 79.8 (5328.6) | 53.5 (42, 68.2) | 171522287 (134570765.6) |
| Diarrhoea | 209 | 16.9 (16.2, 17.6) | 16 (34442.1) | 66.3 (63.6, 69) | 28840442751.9 (27686806005.8) | Haemorrhage intracranial | 3 | 14.6 (5.5, 39.1) | 14.6 (50.6) | 47.7 (17.9, 127.4) | 31600944.1 (11835760.8) |
| Pneumonia | 434 | 15.1 (14.4, 15.8) | 14.5 (22498.9) | 65.5 (62.5, 68.7) | 26202575533.4 (24991965527.3) | Splenomegaly | 3 | 14.4 (7.2, 29) | 14.4 (99.4) | 49.7 (24.8, 99.7) | 31104654.6 (15517935.9) |
| Atrial fibrillation | 244 | 15.9 (15.2, 16.6) | 15.1 (26925.9) | 65.8 (63, 68.8) | 27366161329.8 (26174651006.2) | COVID-19 | 13 | 13.7 (10, 18.7) | 13.3 (468.7) | 54.6 (40, 74.4) | 28888922.7 (21174510.8) |
| Infection | 101 | 12.5 (11.5, 13.6) | 12.4 (5459.4) | 62.2 (57, 67.8) | 22535290720.4 (20672803502.6) | Abdominal distension | 4 | 13.6 (8, 23) | 13.5 (161.5) | 51.4 (30.4, 87.1) | 29151677.7 (17215565.5) |
| Pleural effusion | 106 | 9.6 (9, 10.3) | 9.5 (6222.4) | 63.8 (59.5, 68.4) | 17418492264.5 (16255201410.8) | Lactic acidosis | 3 | 13.5 (6, 30) | 13.4 (68.8) | 49 (22, 109.3) | 29006997.4 (13003128.5) |
| Cardiac failure | 92 | 7.9 (7.1, 8.7) | 7.8 (2086.3) | 61.6 (55.5, 68.4) | 14441866123.7 (13001307554) | Decreased appetite | 4 | 11.5 (7.4, 18) | 11.4 (189.8) | 52.7 (33.9, 82) | 24683944.2 (15873051.1) |
| Anaemia | 151 | 7.8 (7.3, 8.4) | 7.7 (4314) | 63.8 (59.3, 68.6) | 14213227868.9 (13216842286.1) | Diarrhoea | 3 | 11 (8.5, 14.2) | 10.6 (521.9) | 56 (43.2, 72.5) | 22901903.3 (17685251.2) |
| Haemorrhage | 104 | 7.5 (7, 7.9) | 7.3 (5618.9) | 64.8 (60.9, 68.9) | 13528350584.5 (12716856085.1) | Infection | 5 | 10.5 (6.4, 17.2) | 10.4 (136.2) | 52.2 (31.9, 85.4) | 22548198.1 (13773021) |
| Pyrexia | 120 | 5.7 (5.4, 6.1) | 5.6 (3574.9) | 64.9 (60.8, 69.2) | 10442557431.7 (9788676751.1) | Pneumonia | 19 | 10.4 (7.7, 13.9) | 10.1 (377.8) | 55.3 (41.2, 74.2) | 21858506.9 (16294592.3) |
| Septic shock | 96 | 5.7 (4.8, 6.8) | 5.7 (500.6) | 59.2 (49.8, 70.3) | 10562704475.2 (8890845547.3) | Feeding disorder | 3 | 10.4 (5.2, 20.8) | 10.3 (67.2) | 50.2 (25.1, 100.6) | 22303141.1 (11128503.2) |
| Respiratory failure | 166 | 5 (4.4, 5.6) | 5 (801) | 61.3 (54.2, 69.3) | 9240831964.6 (8166981450.9) | Dysphagia | 3 | 8.5 (4.8, 15) | 8.4 (78.5) | 51.7 (29.3, 91.2) | 18237820.9 (10330824.1) |
| Fatigue | 137 | 4.6 (4.5, 4.8) | 4.4 (7542.6) | 68.4 (65.8, 71.1) | 8227546995 (7919418945.4) | Pharyngitis | 3 | 7.2 (2.7, 19.1) | 7.1 (21.1) | 48.7 (18.3, 130.1) | 15478833.9 (5799566.8) |
| Dyspnoea | 117 | 4.6 (4.3, 4.9) | 4.5 (2808.9) | 65.4 (61.5, 69.6) | 8431251251.9 (7923577348.9) | Nausea | 4 | 7.1 (5.4, 9.3) | 6.9 (277.3) | 56.4 (43, 73.8) | 14881527.1 (11366414.5) |
| Asthenia | 139 | 3.9 (3.7, 4.1) | 3.8 (3080.9) | 66.8 (63.5, 70.3) | 7040044719.1 (6687048580.1) | Fall | 6 | 2 (1.4, 2.9) | 2 (15.3) | 56.4 (39.3, 80.9) | 4348087.7 (3028752.9) |
| Fall | 181 | 3.5 (3.3, 3.7) | 3.4 (2556.7) | 66.9 (63.5, 70.5) | 6397063363.8 (6073957153.7) | Dyspnoea | 6 | 6.3 (4.7, 8.4) | 6.1 (206.8) | 56.1 (42.1, 74.8) | 13266021.9 (9949165.8) |
| Thrombocytopenia | 112 | 3.1 (2.8, 3.4) | 3.1 (667.1) | 63.8 (58.3, 69.8) | 5739938760 (5245422883.3) | Pleural effusion | 5 | 6.2 (3.9, 9.8) | 6.1 (82) | 53.4 (34, 84.1) | 13306522.3 (8461522.6) |
| Sepsis | 240 | 3 (2.8, 3.3) | 3 (687.7) | 64.1 (58.7, 69.9) | 5599402037.6 (5134849031) | Haemoglobin decreased | 3 | 4.9 (3.2, 7.4) | 4.8 (69.8) | 54.4 (36, 82.1) | 10432829 (6909786.4) |
| Myocardial infarction | 111 | 1.7 (1.5, 1.8) | 1.7 (108.2) | 64.2 (58.3, 70.8) | 3123146671.7 (2834010860.7) | Fatigue | 5 | 3.8 (3, 4.7) | 3.6 (152.7) | 58.4 (46.6, 73.1) | 7805956.3 (6230793.7) |
| Cardiac arrest | 112 | 1.5 (1.3, 1.7) | 1.5 (31) | 62 (53.6, 71.7) | 2837770790.7 (2452478259.9) | Asthenia | 4 | 2.9 (2.2, 4) | 2.9 (50.1) | 56.7 (41.4, 77.6) | 6275295.4 (4583089.9) |
| Abbreviations: N: the number of reports of BTK-associated AEs; ROR: reporting odds ratio; CI: confidence interval; PRR: proportional reporting ratio; χ2 : chi-squared; BTK：Bruton's tyrosine kinase inhibitor; FAERS:the FDA's Adverse Event Reporting System; | | | | | | | | | | | |

**Figure legends**

**Supplementary Figure 1** SOC distribution of the top 20 AEs of BTK inhibitors according to the number of death reports (A) SOC distribution of ibrutinib-associated AEs according to the number of death reports. (B) SOC distribution of acalabrutinib-associated AEs according to the number of death reports. AEs, adverse effects, SOC, the System Organ Class
